# Linear-Time MaxCut in Multigraphs Parameterized Above the Poljak-Turzík Bound

**DOI:** 10.1007/s00453-025-01306-y

**Published:** 2025-04-10

**Authors:** Jonas Lill, Kalina Petrova, Simon Weber

**Affiliations:** 1 Department of Computer Science, ETH Zürich, Rämistrasse 101, 8092 Zurich, Switzerland; 2 Institute of Science and Technology Austria (ISTA), Am Campus 1, 3400 Klosterneuburg, NÖ Austria

**Keywords:** Fixed-parameter tractability, Maximum cut, Edwards-Erdös bound, Poljak-Turzík bound, Multigraphs, Integer-weighted graphs

## Abstract

MaxCut is a classical $\textsf{NP}$-complete problem and a crucial building block in many combinatorial algorithms. The famous *Edwards-Erdös bound* states that any connected graph on *n* vertices with *m* edges contains a cut of size at least $\frac{m}{2}+\frac{n-1}{4}$. Crowston, Jones and Mnich [Algorithmica, 2015] showed that the MaxCut problem on simple connected graphs admits an FPT algorithm, where the parameter *k* is the difference between the desired cut size *c* and the lower bound given by the Edwards-Erdös bound. This was later improved by Etscheid and Mnich [Algorithmica, 2017] to run in parameterized linear time, i.e., $f(k)\cdot O(m)$. We improve upon this result in two ways: Firstly, we extend the algorithm to work also for *multigraphs* (alternatively, graphs with positive integer weights). Secondly, we change the parameter; instead of the difference to the Edwards-Erdös bound, we use the difference to the *Poljak-Turzík bound*. The Poljak-Turzík bound states that any weighted graph *G* has a cut of weight at least $\frac{w(G)}{2}+\frac{w_{MSF}(G)}{4}$, where *w*(*G*) denotes the total weight of *G*, and $w_{MSF}(G)$ denotes the weight of its minimum spanning forest. In connected simple graphs the two bounds are equivalent, but for multigraphs the Poljak-Turzík bound can be larger and thus yield a smaller parameter *k*. Our algorithm also runs in parameterized linear time, i.e., $f(k)\cdot O(m+n)$.

## Introduction

The $\textsc {MaxCut}$ problem is the problem of deciding whether a given graph *G* contains a cut of size at least as large as a given integer *c*. It has been known for a very long time that this problem is $\textsf{NP}$-complete, in fact it was one of Karp’s 21 $\textsf{NP}$-complete problems [9]. The MaxCut problem has been intensely studied from various angles such as random graph theory and combinatorics, but also approximation and parameterized complexity. It has numerous applications in areas such as physics and circuit design; for more background on the MaxCut problem we refer to the excellent survey by Poljak and Tuza [14].

There are many lower bounds on the maximum cut size $\mu (G)$ of a given graph *G*. If *G* is a graph with *m* edges, a trivial lower bound is $\mu (G)\ge \frac{m}{2}$. This can be shown easily using the probabilistic method, as first done by Erdös [4]. Clearly, $\textsc {MaxCut} (G,c)$ is thus easily solvable if $c\le \frac{m}{2}$. But what if *c* is larger? At which point does the MaxCut problem become difficult? It turns out that already $c=\frac{m}{2}+\epsilon m$ for any fixed $\epsilon >0$ makes the problem $\textsf{NP}$-hard [7]. However, as long as the difference $c-\frac{m}{2}$ is just a constant, $\textsc {MaxCut} (G,c)$ is still polynomial-time solvable: Mahajan and Raman showed in 1999 [11] that $\textsc {MaxCut} (G,\frac{m}{2}+k)$ is fixed-parameter tractable (FPT), i.e., it can be solved in time $f(k)\cdot n^{O(1)}$. This started off the study of *parameterized algorithms above guaranteed lower bounds*.

By the time this FPT algorithm was found, $\frac{m}{2}$ was no longer the best-known lower bound for $\mu (G)$. Already more than 20 years earlier, Edwards showed the following lower bound that was previously conjectured by Erdös, and is thus now known as the Edwards-Erdös bound.

### Theorem 1

(Edwards-Erdös bound [2, 3]) For any connected simple graph *G* with *n* vertices and *m* edges, $\mu (G)\ge \frac{m}{2}+\frac{n-1}{4}$.

Unlike the previous bound of $\frac{m}{2}$, this bound is tight for an infinite class of graphs, for example the odd cliques. It remained open for quite a while whether $\textsc {MaxCut} (G,\frac{m}{2}+\frac{n-1}{4}+k)$ would also be fixed-parameter tractable, i.e., whether the parameter *k* could be reduced by $\frac{n-1}{4}$ compared to the previous result by Mahajan et al. This question was answered in the positive by Crowston, Jones and Mnich, who proved the following theorem.

### Theorem 2

(Crowston et al.  [1, Thm. 1]) There is an algorithm that computes, for any connected graph *G* with *n* vertices and *m* edges and any integer *k*, in time $2^{O(k)}\cdot n^4$ a cut of *G* of size at least $\frac{m}{2}+\frac{n-1}{4}+k$, or decides that no such cut exists.

This algorithm has later been improved to run in linear time (in terms of *m*) by Etscheid and Mnich [5]. However, they study only the problem of deciding the existence of such a cut. An algorithm for computing a cut if one exists could be obtained by adding backtracking to their algorithm, however we believe that like our result (Theorem 5), this would then run in time $2^{O(k)}\cdot n\cdot m$, and not parameterized linear time.

We would like to highlight another classic lower bound on the size of the maximum cut of a graph, nicknamed the “spanning tree” bound: Any connected graph on *n* vertices has a cut of size at least $n-1$, since it contains a spanning tree of this size and trees are bipartite. Note that this bound is incomparable to the Edwards-Erdös bound. In 2018, Madathil, Saurabh, and Zehavi [10] showed that $\textsc {MaxCut} (G,n-1+k)$ is also fixed-parameter tractable.

In 1986, Poljak and Turzík improved upon the Edwards-Erdös bound by replacing the term $n-1$ with the weight of the minimum spanning tree (or forest in disconnected graphs), thus obtaining the following lower bound for maximum cuts in weighted graphs.

### Theorem 3

(Poljak-Turzík bound [13]) For any graph $G=(V,E)$ with weight function $w:E\rightarrow \mathbb {R} _{>0}$, we have $\mu (G)\ge \frac{w(G)}{2}+\frac{w_{MSF}(G)}{4}$, where $w(G)=\sum _{e\in E}w(e)$ and $w_{MSF}(G)$ denotes the weight of a minimum-weight spanning forest of *G*.

It is easy to see that Theorem 3 implies the bound in Theorem 1 both for (unweighted) simple graphs and multigraphs. In unweighted simple graphs it is actually equivalent to Theorem 1, while on multigraphs and positive integer-weighted graphs it can be strictly larger.

The authors of Theorem 2 thus posed as their major open question whether their algorithm could be extended to solve $\textsc {MaxCut} (G,\frac{m}{2}+\frac{n-1}{4}+k)$ on multigraphs as well. We answer this question in the positive, and improve the result further by replacing the Edwards-Erdös bound with the Poljak-Turzík bound.

### Results

We provide a parameterized linear time algorithm for deciding MaxCut in multigraphs and positive integer-weighted (simple) graphs above the Poljak-Turzík bound. A multigraph can be easily turned into a positive integer-weighted graph and vice versa; in the rest of this paper we phrase all of our results and proofs in terms of positive integer-weighted graphs for better legibility.

#### Theorem 4

There is an algorithm that decides for any graph $G=(V,E)$ with weight function $w:E\rightarrow \mathbb {N}$ and any integer *k*, in time $2^{O(k)}\cdot O(|E|+|V|)$, whether a cut of *G* of weight at least $\frac{w(G)}{2}+\frac{w_{MSF}(G)}{4}+k$ exists.

Using the same techniques we can also get a parameterized quadratic-time algorithm to compute such a cut, if one exists.

#### Theorem 5

There is an algorithm that computes for any graph $G=(V,E)$ with weight function $w:E\rightarrow \mathbb {N}$ and any integer *k*, in time $2^{O(k)}\cdot O(|E|\cdot |V|)$, a cut of *G* of weight at least $\frac{w(G)}{2}+\frac{w_{MSF}(G)}{4}+k$, if one exists.

We would like to point out that Theorem 4 is a strict improvement on the linear-time algorithm of Etscheid and Mnich [5] in two ways: Firstly we increase the types of graphs the algorithm is applicable to, and secondly we also strictly decrease the parameter for some instances. The following observation shows that this decrease of parameter can be significant.

#### Observation 6

There exist sequences of positive-integer-weighted graphs $(G_i)_{i\in \mathbb {N}}$ and integers $(c_i)_{i\in \mathbb {N}}$ such that Theorem 4 yields a polynomial-time algorithm to solve $\textsc {MaxCut} (G_i,c_i)$, but when replacing $w_{MSF}(G)$ by $n-1$, it does not.

#### Proof

Let $G_i$ be a tree on $i+1$ vertices where each edge has weight 2. Then, the Poljak-Turzík bound yields $\mu (G_i)\ge \frac{2i}{2}+\frac{2i}{4}=\frac{6}{4}i$, while the Edwards-Erdös bound only yields $\mu (G_i)\ge \frac{2i}{2}+\frac{i+1-1}{4}=\frac{5}{4}i$. Thus, if we set $c_i=\frac{6}{4}i+k$ for some constant *k*, then Theorem 4 yields a $2^{O(k)}\cdot poly(i)=poly(i)$ algorithm for $\textsc {MaxCut} (G_i,c_i)$, while with the Edwards-Erdös bound it would yield a $2^{O(k+\frac{1}{4}i)}\cdot poly(i)$ algorithm, which is not polynomial. $\square$

### Algorithm Overview

Our algorithm works in a very similar fashion to the one in [1]. We use a series of reduction rules that can reduce the input graph down to a graph with no edges. While performing this reduction, we either prove that *G* has a cut of the desired weight, or we collect a set *S* of *O*(*k*) vertices such that $G-S$ is a uniform-clique-forest, i.e., a graph in which every biconnected component is a clique in which every edge has the same weight. Given such a set *S*, we can then compute the maximum cut of *G* exactly: We iteratively test all possibilities of partitioning the vertices in *S* between the two sides of the cut, and then compute the maximum cut of *G* assuming that the vertices of *S* are indeed partitioned like this. To do this, we use a similar approach as in [1]: We compute the maximum cut of $G-S$ with *weighted vertices*. In this setting, each vertex *v* in $G-S$ specifies a weight $w_0(v)$ and $w_1(v)$ for both possible sides of the cut *v* may land in. The value of a cut is given by the total weight of the cut edges plus the sum of the correct weight for each vertex. To use this problem to compute the maximum cut of *G*, we set the weights of each vertex *v* in $G-S$ according to the total weight of the edges between *v* and *S* that are cut in the assumed partition of *S*. Maximizing over all possible partitions for *S* gives the maximum cut of *G*.

While we use very similar techniques as Crowston et al. and Etscheid and Mnich [1, 5], our main technical contribution lies in the reduction rules. Our reduction rules have to be more specific, i.e., each reduction rule has a stronger precondition. This is due to the fact that when performing any reduction, the change in the weight of a minimum spanning forest (as needed for the Poljak-Turzík bound) is much more difficult to track than the number of vertices in the graph (as needed for the Edwards-Erdös bound). Since our rules are more specific, we also need twice as many rules as Crowston et al. [1] (and one more rule than Etscheid and Mnich [5]) to ensure that always at least one rule is applicable to a given graph.

## Preliminaries

In the rest of this paper we consider every graph to be a simple graph $G=(V,E)$, where *V* is the set of vertices, and $$E\subseteq \left( {\begin{array}{c}V\\ 2\end{array}}\right) $$ is the set of edges. A graph is weighted if it is equipped with a positive integer edge-weight function $w:E\rightarrow \mathbb {N}$. For any two disjoint subsets $A,B\subseteq V$ we denote by *E*(*A*, *B*) the set of edges between *A* and *B*, by *w*(*A*, *B*) the total weight of the edges in *E*(*A*, *B*), and by $\min (A,B)$ the minimum weight of any edge in *E*(*A*, *B*). For a subset $A \subseteq V$, we denote by *N*(*A*) the set of vertices in $V {\setminus } A$ that have a neighbor in *A*.

A *spanning forest* of *G* is the union of one spanning tree per connected component of *G*. We denote the minimum weight of all spanning forests of *G* by $w_{MSF}(G)$.

A *cut* is a subset $C\subseteq V$, and the *weight* of a cut *C* is the total weight of the edges connecting a vertex in *C* to a vertex in $V\setminus C$, i.e., $w(C)=w(C,V\setminus C)$.

For any set $A\subseteq V$ we write *G*[*A*] for the graph on *A* induced by *G*, and $G-A$ for the graph on $V\setminus A$ induced by *G*.

We say that a graph is *uniform* if all of the edges have the same weight. More specifically, we call a graph *c*-uniform if all edges have weight *c*.

A graph (*V*, *E*) is called *biconnected*, if $|V|\ge 1$, and for every vertex $v\in V$, $G-\{v\}$ is connected. A *biconnected component* of a graph is a maximal biconnected subgraph, also referred to as a *block*. It is well-known that the biconnected components of every graph partition its edges. A vertex that participates in more than one biconnected component is a *cut vertex* (usually defined as a vertex whose removal disconnects a connected component). A graph can thus be decomposed into biconnected components and cut vertices.

### Definition 1

(Block-Cut Forest) The *block-cut forest* *F* of a graph *G* has vertex set $V(F) = \mathcal {C} \cup \mathcal {B}$, where $\mathcal {C}$ is the set of cut vertices of *G* and $\mathcal {B}$ is the set of biconnected components of *G*, and $\{B,c\}$ is an edge in *F* if $B \in \mathcal {B}$, $c \in \mathcal {C}$, and $c \in V(B)$.

It is not hard to see that the block-cut forest *F* of a graph *G* is indeed a forest, since a cycle in it would imply a cycle in *G* going through multiple biconnected components, thus contradicting their maximality. Moreover, each connected component of *F* corresponds to a connected component of *G*, and all leaves of *F* are biconnected components in *G*. We refer to the biconnected components of *G* that correspond to leaves of *F* as *leaf-blocks* of *G*.

### Definition 2

(Uniform-Clique-Forest) A weighted graph is a *uniform-clique-forest* if each of its blocks *B* is a uniform clique.

### Definition 3

The problem MaxCut-With-Vertex-Weights is given as follows.

Input: A weighted graph (*V*, *E*) with edge-weight function *w*, as well as two vertex-weight functions $w_0:V\rightarrow \mathbb {N}$, $w_1:V\rightarrow \mathbb {N}$.
Output: A cut *C* maximizing $w(C)+\sum _{v\in C}w_1(v)+\sum _{v\not \in C}w_0(v)$.

We show in Sect. 4 that MaxCut-With-Vertex-Weights is solvable in linear time if the input graph is a uniform-clique-forest.

## Reducing to a Uniform-Clique-Forest

In the first part of our algorithm, we wish to either already conclude that the input graph has a cut of the desired weight, or to find some set *S* of vertices such that $G-S$ is a uniform-clique-forest.

### Lemma 7

For any graph $G=(V,E)$ on *n* vertices with *m* edges and weight function $w:E\rightarrow \mathbb {N}$ and any integer *k*, in time $O(n+k\cdot m)$ one can either decide that *G* has a cut of weight at least $\frac{w(G)}{2}+\frac{w_{MSF}(G)}{4}+\frac{k}{4}$, or find a set $S\subseteq V$ such that $|S|\le 3k$ and $G-S$ is a uniform-clique-forest.

Note that we write $\frac{k}{4}$ instead of just *k*. The reason for this is that with our reduction rules we make “progress” reducing the difference to the Poljak-Turzík bound in increments of $\frac{1}{4}$.

To prove Lemma 7 we use eight reduction rules, closely inspired by the reduction rules used in previous work [1, 5]. Each reduction rule removes some vertices from the given graph, possibly *marks* some of the removed vertices to be put into *S*, and possibly *reduces* the parameter *k* by 1. To prove Lemma 7, the reduction rules will be shown to fulfill the following properties.

Firstly, each reduction rule ensures a one-directional implication: if the reduced graph $G'$ contains a cut of weight $\frac{w(G')}{2}+\frac{w_{MSF}(G')}{4}+\frac{k'}{4}$ (where $k'$ is the possibly reduced *k*), then the original graph *G* must also contain a cut of weight $\frac{w(G)}{2}+\frac{w_{MSF}(G)}{4}+\frac{k}{4}$. By the Poljak-Turzík bound, if *k* ever reaches 0, it is clear that the original graph *G* must have contained a cut of the desired weight.

Secondly, we need that to every graph with at least one edge, at least one of the rules applies. To get our desired runtime, we also need that an applicable rule can be found and applied efficiently.

Thirdly, every rule should only mark at most three vertices to be added to *S*. If a rule does not reduce *k*, it may not mark any vertices. This ensures that at most 3*k* vertices are added to *S*.

Lastly, we require that after exhaustively applying the rules and reaching a graph with no more edges, the graph $G-S$ is a uniform-clique-forest.

**Fig. 1:**
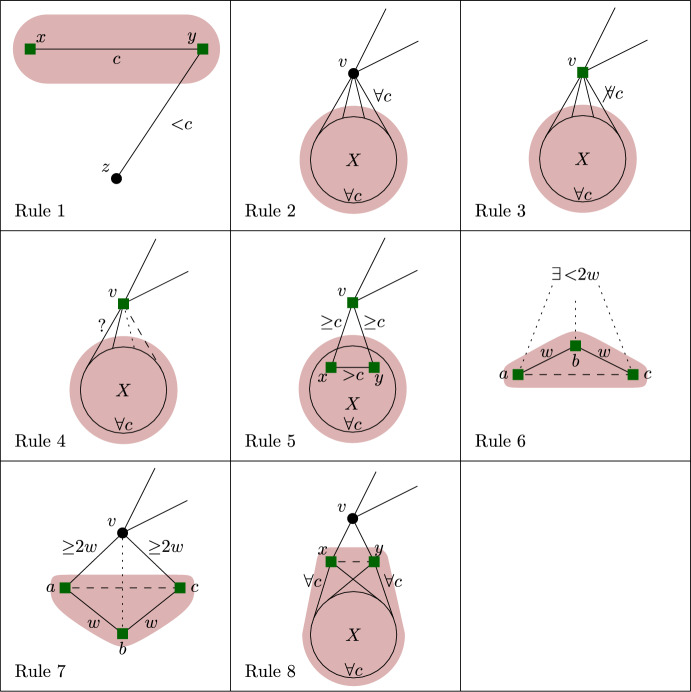
The eight reduction rules. An edge is drawn normally if it must exist for the rule to apply. Some edges are drawn dashed to emphasize that they *must not* exist for the rule to apply. Some additional edges are drawn dotted to emphasize that they *may* exist but do not have to. Red shading indicates the vertices removed by the rule, while vertices marked by the rule are drawn using a green square

We will now state our reduction rules, and then prove these four properties in Lemmas 8 and 9, Observation 10, and Lemma 11, respectively. For simplicity, each reduction rule is stated in such a way that it assumes the input graph to be connected. If the input graph is disconnected, instead consider *G* to be one of its connected components. Each rule preserves connectedness of the connected component it is applied to, which we also show in Lemma 8. Note further that if the connected component the rule is being applied to is also biconnected, then if the precondition requires some vertex to be a cut vertex, any vertex can play that role, although technically there are no cut vertices. We state this once here for simplicity, instead of saying each time that either *v* is a cut vertex or *G* is biconnected. We visualize the eight rules in Fig. 1.
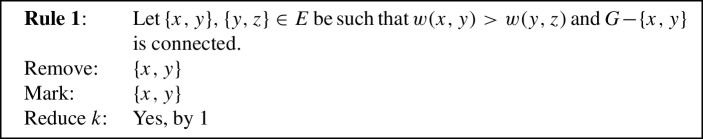

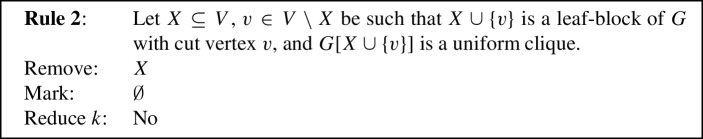

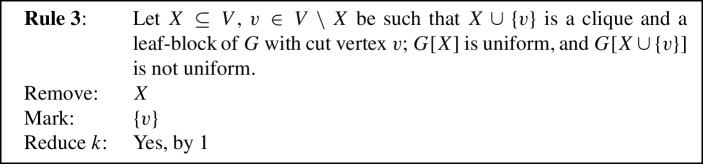

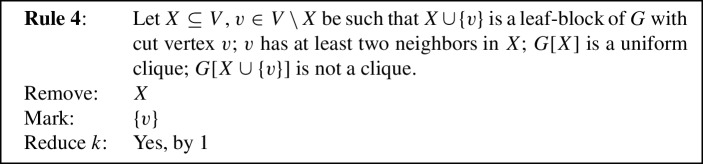

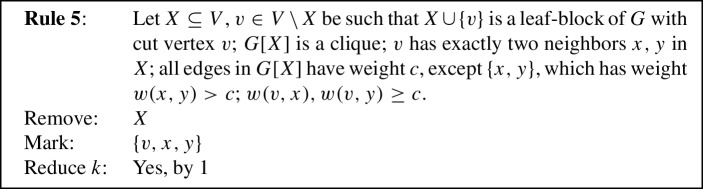

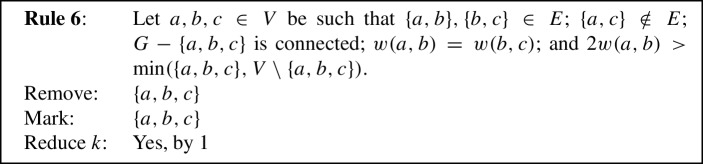

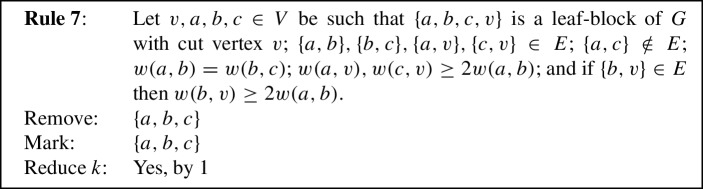

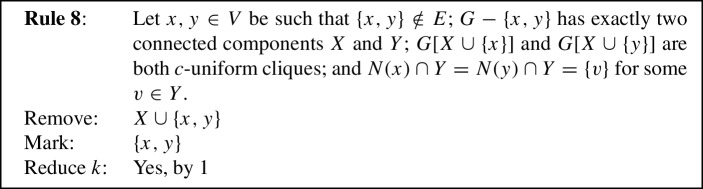

We first state the formalizations of our four properties, then prove Lemma 7, and only then prove each of our properties.

### Lemma 8

Let $G=(V,E)$ be a graph with weights *w*, and let *k* be any positive integer. Let $G'$ be the result of one application of one of the rules 1–8 to *G*, and $k'$ the resulting parameter. Then, if $G'$ has a cut of weight at least $\frac{w(G')}{2}+\frac{w_{MSF}(G')}{4}+\frac{k'}{4}$, then *G* must contain a cut of weight at least $\frac{w(G)}{2}+\frac{w_{MSF}(G)}{4}+\frac{k}{4}$. Furthermore, if *G* is connected, then $G'$ is connected.

### Lemma 9

Let $G=(V,E)$ be a weighted graph with at least one edge. Given the block-cut forest of *G* we can either apply Rule 2 in time $O(|E_X|)$ where $E_X$ is the set of edges removed by applying Rule 2, or we can find and apply another rule in time *O*(|*E*|). In the same time we can also make the necessary changes to the block-cut forest to obtain the block-cut forest of the resulting graph $G'$.

### Observation 10

Each rule marks at most three vertices. Rule 2, the only rule that does not reduce *k*, does not mark any vertices.

### Lemma 11

Let *S* be the set of vertices marked when exhaustively (i.e., until *G* has no edges) applying Rules 1–8 to a graph *G*. Then $G - S$ is a uniform-clique-forest.

Let us now prove Rule 7 using these properties.

### Proof of Lemma 7

We begin by computing the block-cut forest of *G* in $O(n+m)$ time [8]. Then, we apply rules until we either reach $k=0$ or until we reach a graph with no edges. Whenever we apply a rule, we locally adapt the block-cut forest. In total we apply rules other than Rule 2 at most *k* times. By Lemma 9 this takes at most $O(k\cdot m)$ time. Since applying Rule 2 takes time $O(|E'|)$ where $E'$ is the set of edges removed, all applications of Rule 2 together use time *O*(*m*). The reduction step can thus be performed in $O(k\cdot m)$.

If we have reached $k=0$, by the Poljak-Turzík bound and by Lemma 8 we can decide that our input graph contains a cut of the desired weight. Otherwise, by Observation 10, *S* contains at most 3*k* vertices. By Lemma 11, $G-S$ then forms a uniform-clique-forest, and we have proven our desired statement. $\square$

We will now proceed to prove Lemmas 8,9 and 11. The main technical challenges are the proofs of Lemmas 8 and 9. These proofs are more technically involved than the corresponding proofs by Etscheid and Mnich [5]. For Lemma 8 this is due to the fact that the weight of a minimum spanning forest is much more difficult to track through a reduction than the number of vertices. For Lemma 9 the proof is more involved since our rules are more specific, and thus more case distinction is needed. We present the proof of Lemma 8 in Section A, since despite its technicality, it is not very insightful.

To prove Lemma 9 we use the following lemma, the proof of which follows from the proof of [5, Lemma 3] rather directly.

### Lemma 12

Let $G=(V,E)$ be a connected graph with at least one edge, and let $B\subseteq V$ be a biconnected component that is a leaf in the block-cut forest of *G*. Now, we write *B* as $X\cup \{v\}$, where *v* is the cut vertex disconnecting $B=X\cup \{v\}$ from $V\setminus B$ (if *B* is an isolated vertex in the block-cut forest, i.e., it forms a connected component of *G* that is also biconnected, then let *v* be an arbitrary vertex in *B*). Then at least one of the following properties holds.

A) $$G[X\cup \{v\}]$$ is a clique.
B) *G*[*X*] is a clique but $G[X\cup \{v\}]$ is not a clique.
C) *v* has exactly two neighbors in *X*, *x* and *y*. Furthermore, $\{x,y\} \notin E$, and $G[X{\setminus }\{x\}]$ and $G[X\setminus \{y\}]$ are cliques.
D) $X\cup \{v\}$ contains vertices *a*, *b*, *c* such that $\{a,b\},\{b,c\} \in E$, $\{a,c\}\notin E$, and $G - \{a,b,c\}$ is connected.

Furthermore, such a property (including the vertices *x*, *y* and *a*, *b*, *c* for cases C and D, respectively) can be found in linear time in the number of edges in *G*[*X*].

### Proof

(sketch) One can check whether *G*[*X*] is a clique for some $X\subseteq V$ in time linear in the number of edges in *G*[*X*]. To do this, we simply check whether each edge is present in some fixed order. It is thus easy to check for cases A), B), and C) in linear time.

In the proof of [5, Lemma 3] it is shown that if none of the cases A), B), and C) apply, then vertices *a*, *b*, *c* certifying case D) can be found in linear time. $\square$

### Proof of Lemma 9

Without loss of generality we can assume that *G* is connected; otherwise, we consider *G* to be an arbitrary connected component of our input graph that contains at least one edge. We first apply Lemma 12 on a leaf-block $X\cup \{v\}$ to find one of the four properties.

***Property A*** If property A holds, we can check whether $G[X\cup \{v\}]$ is uniform in time $O(|E'|)$ where $E'$ is the set of edges in $G[X\cup \{v\}]$. In this process we can track also whether *G*[*X*] is uniform. If $G[X\cup \{v\}]$ is uniform we apply Rule 2. Else, if only *G*[*X*] is uniform, we apply Rule 3. If not even *G*[*X*] is uniform, we can find two edges $\{x,y\},\{y,z\}$ in *G*[*X*] such that $w(x,y)>w(y,z)$. Since $X\cup \{v\}$ is a clique, $G-\{x,y\}$ must be connected. We can therefore apply Rule 1.

***Property B*** We can handle property B in a similar way. If *G*[*X*] is uniform, we can apply Rule 4. Else, we apply case distinction on the number of vertices in *X* adjacent to *v*. We first consider the case if vertex *v* is adjacent to exactly two vertices in *X*. Since *X* is not uniform, there exist vertices $x,y \in X$ and a vertex $u\in X\cup \{v\}$ such that $w(x,y) > w(x,u)$. If the only such choice of *x*, *y* is such that *x* and *y* are exactly the two vertices in *X* adjacent to *v*, then we can apply Rule 5. Else we can see that $G - \{x,y\}$ must be connected and apply Rule 1. Let us now consider the other case, that vertex *v* is adjacent to at least three vertices in *X*. There must again exist vertices $u,x,y \in X$ so that $w(x,y) > w(x,u)$. Since *v* is adjacent to at least three vertices and *G*[*X*] is a clique, $G - \{x,y\}$ is connected and we can apply Rule 1.

***Property C*** To handle Property C we first check whether *G*[*X*] is uniform. If it is not, we can apply Rule 1, since for any edge $\{a,b\}$ in *G*[*X*], $G-\{a,b\}$ is connected. Knowing that *G*[*X*] is uniform, and that *v* has exactly two neighbors, we can apply Rule 8.

***Property D*** Note that since $G - \{a,b,c\}$ is connected, and since by its biconnectedness $B\ne \{a,b,c\}$, if $G - B$ is non-empty, then $v \notin \{a,b,c\}$. Next, again since *G*[*B*] is biconnected, we must have the following. $\square$

### Assumption 1

We have $E(\{a\},B\setminus \{a,b,c\})\ne \emptyset$ and $E(\{c\},B\setminus \{a,b,c\})\ne \emptyset$.

From this we get that $G-\{a,b\}$ and $G-\{b,c\}$ must be connected. Thus, we can compare *w*(*a*, *b*) and *w*(*b*, *c*), and apply Rule 1 if $w(a,b)\ne w(b,c)$. We can also compute the value $m=\min (\{a,b,c\},B\setminus \{a,b,c\})$. If $2w(a,b)>m$ we can apply Rule 6. Thus, we can make the following assumptions.

### Assumption 2

We can assume $w(a,b)=w(b,c)$.

### Assumption 3

We can assume $2w(a,b) \le \min (B- \{a,b,c\}, \{a,b,c\})$.

Next, we compute the block-cut forests for the four graphs $G_{abc}:=G[B]-\{a,b,c\}$ and $G_u:=G[B]-\{u\}$ for all $u\in \{a,b,c\}$. This can be performed in the required time, and yields the set of cut vertices for all these graphs. We now test for every $u\in \{a,b,c\}$ and for every vertex $z\in B {\setminus } \{a,b,c\}$ with $\{z,u\}\in E$ whether *z* is a cut vertex in $G_u$. If for any such pair *z*, *u* we have that *z* is *not* a cut vertex in $G_u$, this means that $G-\{u,z\}$ is connected because if $G - B$ is non-empty, $v \notin \{a,b,c\}$; we can thus apply Rule 1 to that edge (since by Assumptions 2 and 3, every edge in $E(\{a,b,c\},B\setminus \{a,b,c\})$ has weight at least twice as large as $w(a,b)=w(b,c)$). From now on, we can make the following assumption.

### Assumption 4

For every pair of vertices $z \in B {\setminus } \{a,b,c\}$ and $u\in \{a,b,c\}$ with $\{z,u\}\in E$, *z* is a cut vertex of $G_{u}$.

We now check whether any vertex $z\in B \setminus \{a,b,c\}$ that is adjacent to some $u\in \{a,b,c\}$ is *not* a cut vertex in $G_{abc}$.

Case 1: There is a vertex $z\in B\setminus \{a,b,c\}$ adjacent to some $u\in \{a,b,c\}$ such that *z* is a cut vertex of $G_u$ but not of $G_{abc}$. We claim that we can apply Rule 7. We prove this by distinguishing two cases, depending on *u*:

- $u\in \{a,c\}$: Suppose without loss of generality that $u=a$. Since *z* is a cut vertex of $G_a$, it follows that $G_a - z$ has $t\ge 2$ connected components $C_1, \dots , C_t$. Suppose without loss of generality that $b,c \in C_1$. If $C_1 {\setminus } \{b,c\} \ne \varnothing$, then $C_1 {\setminus } \{b,c\}$ and $C_2$ lie in different connected components of $G_{abc} - z$, implying that *z* is a cut vertex of $G_{abc}$, which contradicts the assumption of Case 1. Thus $C_1 = \{b,c\}$, implying that *b* and *c* have no neighbours in $B \setminus \{a,b,c,z\}$. Therefore $\{c,z\} \in E$ by Assumption 1, so *c* can also play the role of *u*. By symmetry, *a* has no neighbours in $B {\setminus } \{a,b,c,z\}$ and $\{a,z\} \in E$. It follows that $\{a,b,c,z\}$ is a leaf-block of *G* and so Rule 7 applies.
- $u=b$: Let $S:= B \setminus \{a,b,c,z\}$. Recall that by Assumption 1, $E(\{a\},S \cup \{z\})$ and $E(\{c\}, S \cup \{z\})$ are both non-empty. We will show that either $\{a,z\} \in E$ or $\{c,z\} \in E$ (or both hold). Suppose that is not the case. Then $E(\{a\},S)$ and $E(\{c\}, S)$ are both non-empty. Since *z* is not a cut vertex in $G_{abc}$ (as we are in Case 1), the graph *G*[*S*] must be connected. That implies $G[S \cup \{a,c\}] = G[B] - \{b,z\}$ is also connected, which contradicts our assumption that *z* is a cut vertex of $G_b$ (Assumption 4). We have shown that at least one of $\{a,z\}$ and $\{c, z \}$ is in *E*, say $\{a,z\}$. Thus, without loss of generality, the case $u \in \{a,c\}$ applies, since *z* must be a cut vertex of $G_a$ by Assumption 4. We have thus reduced the case $u=b$ to $u \in \{a,c\}$, which we already handled.

Case 2: There is no vertex $z\in B\setminus \{a,b,c\}$ adjacent to some $u\in \{a,b,c\}$ such that *z* is a cut vertex of $G_u$ but not of $G_{abc}$. Together with Assumption 4, this implies we can assume the following.

### Assumption 5

For every vertex $z \in B {\setminus } \{a,b,c\}$ that has a neighbor in $\{a,b,c\}$, we have that *z* is a cut vertex of $G_{abc}$.

One can now show that if this point is reached without having found an applicable rule, then Rule 7 must be applicable to the graph. We will use the following claim that we will prove later.

### Claim 13

Let *G* be a connected graph with $X \subset G$ where *X* and $G - X$ are connected, and for all $v \in V(G - X)$ it holds that if *v* has a neighbor in *X*, then *v* is a cut vertex of $G - X$. If $|N(X)| \ge 2$, then there are two distinct vertices $v_1,v_2 \in N(X)$ that are both cut vertices of *G*.

We apply Claim 13 on the set $X:=\{a,b,c\}$ and the graph *G*, which we can do by Assumption 5. Observe that since *B* is biconnected containing at most one cut vertex of *G*, it follows that $N(\{a,b,c\})$ contains at most one cut vertex of *G*. This, together with Assumptions 4 and 5 and Claim 13, implies that $|N(\{a,b,c\})| = 1$. The vertex in $N(\{a,b,c\})$ must be the cut vertex *v*, as removing it would disconnect $\{a,b,c\}$ from the rest of *G*. By Assumption 1, we know that $\{a,v\}, \{c,v\} \in E$. By Assimptions 2 and 3, all the weight restrictions of Rule 7 are satisfied, which can thus be applied. $\square$

### Proof of Claim 13

Let *H* be the block-cut forest of $G-X$ and suppose $V(H) = \mathcal {C} \cup \mathcal {B}$, where $\mathcal {C}$ are the cut vertices of $G-X$ and $\mathcal {B}$ are the biconnected components of $G-X$. Since $|N(X)| \ge 2$, we get that $|\mathcal {C}| \ge 2$. Note that all leaves of *H* are in $\mathcal {B}$. Consider the forest *F* that we obtain by removing all leaves of *H*, and note that *F* has at least two vertices since $\mathcal {C} \subseteq V(F)$. Thus, *F* has at least two leaves, say $\ell _1, \ell _2$, each of which must be in $\mathcal {C}$, since its neighbors in $H\setminus F$ are in $\mathcal {B}$. Let $B' \in \mathcal {B}$ be a leaf of *H* that is a neighbor of $\ell _i$ for some $i\in \{1,2\}$. Since every vertex in *N*(*X*) is in $\mathcal {C}$, it follows that $E(X, B'\setminus \{\ell _i\}) = \varnothing$, so $\ell _i$ is a cut vertex in *G*. $\square$

For this section, it only remains to prove Lemma 11.

### Proof of Lemma 11

Let $G_1,G_2,\ldots ,G_q$ be the sequence of graphs obtained while exhaustively applying rules 1–8 to $G_1$ ($G_2$ is the graph obtained after applying one rule to $G_1$, $G_3$ is the graph obtained after applying one rule to $G_2$, and so on). We prove that for any graph $G_i$ in the sequence, $G_i-S$ is a uniform-clique-forest. We run this proof by induction over the sequence of graphs in reverse order (in the order $G_q,G_{q-1}$,...,$G_2$,$G_1$).

*Base Case:* By Lemma 9, we know that $G_q$ is a graph without edges, therefore $G_q - S$ is trivially a uniform-clique-forest.

*Induction Hypothesis: * Assume $G_i - S$ is a uniform-clique-forest.

*Step Case:* We prove that $G_{i-1} - S$ is a uniform-clique-forest. We know that one rule among rules 1–8 was applied to $G_{i-1}$ to obtain $G_i$. We do a case distinction over which rule was applied:

- Rule 1, 6, or 7 was applied to $G_{i-1}$. Every vertex these rules remove is also marked, therefore $G_{i-1} - S = G_i - S$.
- Rule 2 was applied to $G_{i-1}$. We can create $G_{i-1}-S$ from $G_i-S$ by connecting a clique *X* to a vertex $v \in V(G_i)$ such that $X \cup \{v\}$ is a uniform clique. If *v* is in *S*, this is instead adding a disjoint uniform clique. Observe that this just adds a uniform leaf-clique in either case.
- Rule 3, 4, 5, or 8 was applied to $G_{i-1}$. We can create $G_{i-1}-S$ from $G_i-S$ by adding a disjoint uniform clique.

We conclude that in all cases $G_{i-1} - S$ consists of one or zero uniform cliques added to $G_i - S$ as a leaf, and thus by the induction hypothesis $G_{i-1}-S$ is a uniform-clique-forest. $\square$

## Solving MaxCut-With-Vertex-Weights on Uniform-Clique-Forests

### Lemma 14

MaxCut-With-Vertex-Weights on a uniform-clique-forest *G* with *n* vertices and *m* edges can be solved in $O(n + m)$ time.

### Proof

This proof loosely follows the proof of [5, Lemma 4]. We first compute the cut-block forest of *G*. We know that every graph contains at least one leaf-block. Let $X \cup \{v\}$ be a leaf-block of *G* where $v \in V(G)$ is the cut vertex of *X* (if a connected component of *G* consists of a single biconnected component *B*, then $X = B - \{v\}$ where *v* is an arbitrary vertex in *B*). Let $n' = |X|$ and $m'$ be the number of edges in $G[X \cup \{v\}]$. Since *G* is a uniform-clique-forest, we know that $G[X\cup \{v\}]$ is *c*-uniform for some *c*. We now consider the maximum weighted cut *C* in $G[X\cup \{v\}]$ under both possible restrictions $v\not \in C$ and $v\in C$.

We first consider $v\not \in C$. Let $\delta (x) = w_1(x) - w_0(x)$ for every vertex $x \in X$. We can sort the vertices in *X* in the order $x_1,x_2,..,x_{n'}$ with decreasing $\delta$-value, i.e., $\delta (x_1)\ge \ldots \ge \delta (x_{n'})$. For any $p\in \{0,\ldots ,n'\}$, we let $A_p$ be the set $\{x_1,\ldots ,x_p\}$. $A_p$ is the best cut among all cuts $C'$ with $|C' \cap X|=p$, since by uniformity of $G[X\cup \{v\}]$ all such cuts have the same weight $w(C')$, and the value of such a cut $C'$ is equal to $w(C')+\sum _{v\in X}w_0(x) + \sum _{v\in C}(w_1(v)-w_0(v))$. Now we can find the maximum weighted cut in $X\cup \{v\}$ by comparing the $n'+1$ cuts $A_0,..,A_{n'}$. Letting $\lambda$ be the value of this cut, we update $w_0(v) = \lambda$.

We can perform the same process for $v\in C$. We instead consider $A_p=\{v,x_1,\ldots ,x_p\}$, and update $w_1(v)$ to the optimum value found. After having updated both weights for *v*, we can now delete all vertices in *X*.

We can apply this method to *G* exhaustively until we are left with a graph with no edges. The desired value of the maximum weighted cut on the entire graph *G* is the sum of the greater values of $w_0(v)$ or $w_1(v)$ for all remaining vertices *v*.

We now calculate the runtime of this method applied to one leaf-block *X*. Sorting the vertices takes $O(n'\log (n'))$ time. Since *X* is a clique, we have $n'\log (n')\le \frac{n'(n'+1)}{2}=m'$ for all $n'\ge 4$. We can calculate the value of the assignment $A_0$ in $O(m')$ time. Observe that the difference between cuts $A_i$ and $A_{i+1}$ for any $i \in \{0,..,n-1\}$ is in only one vertex. By only considering these local modifications we can calculate the values of the cuts $A_0,..,A_{n'}$ in $O(m')$ time. Since in every iteration we perform this process on a different block, in total we can bound our runtime with $O(n + m)$, since for blocks with $n'<4$ the runtime of $O(n'\log (n'))=O(1)$ can be charged to some vertex in the block, while for blocks with $n'\ge 4$ the runtime of $O(n'\log (n'))$ can be expressed as $O(m')$. $\square$

## Conclusion

With Lemmas 7 and 14, our main result now follows easily:

### Proof of Theorem 4

Given any instance $\textsc {MaxCut} (G,\frac{w(G)}{2}+\frac{w_{MSF}(G)}{4}+\frac{k'}{4})$ with $k':=4k$, by Lemma 7 we can in time $O(n+k\cdot m)$ either decide that the instance is a “yes”-instance, or find a set $S\subseteq V$ with $|S|\le 3k'=12k$ such that $G-S$ is a uniform-clique-forest. For each subset $S'\subseteq S$ we can then in time $O(n+m)$ build a MaxCut-With-Vertex-Weights instance on the graph $G-S$, such that the vertex weights $w_0(v)$ and $w_1(v)$ of a vertex $v\in G-S$ denote the sum of the weights of edges to vertices in $S'$ and $S{\setminus } S'$ respectively. By Lemma 14, each of these instances can be solved in $O(n+m)$ time. The maximum cut found in any instance given by a set $S'$ corresponds to the maximum cut *C* of *G* obtainable under the condition that $C\cap S=S'$. Taking into account the edges between *S* and $S'$ and taking the maximum over all instances thus computes the maximum cut weight of *G*.

To compute the overall runtime, note that since $|S|\le 12k$, we solve at most $2^{12k}$ MaxCut-With-Vertex-Weights instances. Thus, the overall runtime is $O(n+k\cdot m+2^{O(k)}\cdot (n+m))=O(2^{O(k)}\cdot (n+m))$. $\square$

If we want to find a cut instead of deciding the existence of a cut, we can use very similar techniques.

### Proof of Theorem 5

The proof of Lemma 8 is constructive: given a cut $C'$ on the reduced graph $G'$ of the assumed weight, a cut *C* on the original graph *G* of the required weight can be found in linear time in the number of removed edges and vertices. Thus, instead of applying reduction rules only until $k\le 0$ or until the graph has no edges, we *always* apply rules until the graph contains no edges. This requires at most $O(n\cdot m)$ time. Note that when we have removed all edges from the graph, the required weight of a cut (*k* larger than the Poljak-Turzík bound) is simply $\frac{0}{2}+\frac{0}{4}+k=k$. Thus, if $k\le 0$ is reached, the required cut weight is non-positive, thus we can start with any arbitrary cut $C'$ of the remaining independent set. We can then apply the cut extensions from the proof of Lemma 8 for all applied rules in reverse. This yields a cut of *G* of the desired weight. If otherwise we have $k>0$ when we reached a graph with no edges, we know that $|S|\le 12k$, and we can again solve $2^{|S|}$ instances of MaxCut-With-Vertex-Weights on $G-S$. $\square$

### Open Problems

Our result leaves a few interesting open problems.

#### Other $\lambda$-extendible properties

In [13], Poljak and Turzík actually not only show the lower bound for MaxCut (Theorem 3) but in fact they prove a very similar bound for the existence of large subgraphs fulfilling any so-called $\lambda$*-extendible* property.^1^ Mnich, Philip, Saurabh, and Suchý [12] generalize the approach of Crowston et al. [1] for MaxCut to work for a large subset of these $\lambda$-extendible properties. Note that while the title of [12] includes “above the Poljak-Turzík bound”, the authors restrict their attention to unweighted simple graphs, and thus their result applied to MaxCut only implies the result of Crowston et al. [1], but *not* our result. We find it a very interesting direction to see if our result can be extended to also cover some more $\lambda$-extendible properties in multigraphs or positive integer-weighted graphs.

#### Kernelization

Many previous works on MaxCut parameterized above guaranteed lower bounds have also provided kernelization results [1, 5, 10]. In particular, together with their linear-time algorithm parameterized by the distance *k* to the Poljak-Turzík bound, Etscheid and Mnich [5] also provide a linear-sized (in *k*) kernel. We are not aware of any kernelization results for MaxCut on multigraphs or positive integer-weighted graphs. It would thus be very interesting to explore whether these results can also be extended to our setting.

#### FPT above better lower bounds

Recently, Gutin and Yeo [6] proved new lower bounds for $\mu (G)$ for positive real-weighted graphs. In particular, they prove $\mu (G)\ge \frac{w(G)}{2}+\frac{w(M)}{2}$ where *M* is a maximum matching of *G*, and $\mu (G)\ge \frac{w(G)}{2}+\frac{w(D)}{4}$ for any DFS-tree *D* (which implies the Poljak-Turzík bound). Both of these bounds are consequences of a more general bound involving disjoint bipartite induced subgraphs, but the value of this bound is $\textsf{NP}$-hard to compute [6]. The weight of the largest DFS-tree is also $\textsf{NP}$-hard to compute [6]. These two bounds are thus not very suitable for an FPT algorithm, but the bound involving the maximum matching may be, since the maximum matching in a weighted graph can be computed in polynomial time using Edmonds’ blossom algorithm.

#### General weights

After going from simple graphs to multigraphs and thus positive integer-weighted graphs, it would be interesting to further generalize to positive real-weighted graphs. Here, it is not directly clear what the parameter *k* exactly should be. Generalizing our algorithm may require completely new approaches since we cannot discretize the decrease of *k*.

## Data Availability

No datasets were generated or analysed during the current study

## Appendix A Proof of Lemma 8

We will often use the following claim that slightly strengthens the Poljak-Turzík bound in certain cases:

### Claim 15

Let $G=(V,E)$ be a weighted graph with weights $w:E\rightarrow \mathbb {N}$ such that there exist edges $\{u,v\},\{v,x\}$ with $w(u,v) > w(v,x)$ and $G - \{u,v\}$ is connected. Then *G* has a cut of weight at least $\frac{w(G)}{2} + \frac{w_{MSF}(G)}{4} + \frac{1}{4}$.

### Proof

Let $G'$ = $G - \{u,v\}$. By the Poljak-Turzík bound we know we have a cut $C'$ of $G'$ of weight at least $\frac{w(G')}{2} + \frac{w_{MSF}(G')}{4}$. We can extend this to a cut *C* in *G* by adding exactly one of *u* and *v*. We choose the one such that at least half of the weight in $E(\{u,v\},V')$ goes over the cut, where $V' = V - \{u,v\}$. We have that $MSF(G') \cup \{u,v\} \cup \ \{v,x\}$ is a spanning forest of *G*, therefore $w_{MSF}(G') + w(u,v) + w(v,x) \ge w_{MSF}(G)$. The cut *C* has weight at least

$$\begin{aligned} w(C)&\ge \frac{w(G')}{2} + \frac{w_{MSF}(G')}{4} + \frac{w(\{u,v\},V')}{2} + w(u,v)\\&= \frac{w(G)}{2} + \frac{w_{MSF}(G')}{4} + \frac{w(u,v)}{2}\\&\ge \frac{w(G)}{2} + \frac{w_{MSF}(G')}{4} + \frac{w(u,v)}{4} + \frac{w(v,x)+1}{4}\\&\ge \frac{w(G)}{2} + \frac{w_{MSF}(G)}{4} + \frac{1}{4}, \end{aligned}$$

where on the second line we used that $w(G) = w(G') + w(\{u,v\},V') + w(u,v)$, and on the third line we used that $w(u,v) > w(v,x)$. $\square$

Let us now prove Lemma 8.

### Proof of Lemma 8

We first see that each rule preserves connectedness simply by their preconditions. Each rule either explicitly requires that the resulting graph is connected (Rules 1,6 and 8), or removes a whole leaf-block of *G*, except for the cut vertex (Rules 3to 2,7).

We now prove the required cut weight implication for each rule independently. We need to prove that if there exists a cut $C'$ in $G'$ that produces a cut of weight $\frac{w(G')}{2} + \frac{w_{MSF}(G')}{4} + \frac{k'}{4}$, then this can be extended to a cut *C* of *G* of weight $\frac{w(G)}{2} + \frac{w_{MSF}(G)}{4} + \frac{k}{4}$. We thus assume that such a cut $C'$ exists, and then extend it in such a way that $C\cap V' = C'$. We perform a case distinction on the rule that we applied to *G* to obtain $G'$. Recall that for all rules except Rule 2, $k'=k-1$.

***Rule*** 1: We extend $C'$ by putting *x* and *y* on different sides of the cut. Among the two possibilities, we choose the one such that at least half the weight in $E(\{x,y\},V')$ goes over the cut. We get a cut of weight at least

$$\begin{aligned}w(C)&\ge \frac{w(G')}{2} + \frac{w_{MSF}(G')}{4} + \frac{k'}{4} + w(x,y) + \frac{w(E(\{x,y\},V'))}{2} \\&= \frac{w(G)}{2} + \frac{w_{MSF}(G')}{4} + \frac{k'}{4} + \frac{w(x,y)}{2}\\&\ge \frac{w(G)}{2} + \frac{w_{MSF}(G')}{4} + \frac{k'}{4} + \frac{w(x,y)}{4} + \frac{w(y,z)+1}{4}, \end{aligned}$$

where we used that $w(G) = w(G') + w(E(\{x,y\},V')) + w(x,y)$ in the second step, and that $w(x,y) > w(y,z)$ in the third step. We now see that $w_{MSF}(G)\le w_{MSF}(G')+w(x,y)+w(y,z)$, and we thus get

$$\begin{aligned}w(C)\ge \frac{w(G)}{2} + \frac{w_{MSF}(G)}{4}+\frac{k}{4}.\end{aligned}$$

***Rule*** 2: We can assume without loss of generality that $v\in C'$. Let $n':= |X \cup \{v\}|$. Observe that the sum of the total weight in $G[X\cup \{v\}]$ is $c\left( \frac{n'(n'-1)}{2}\right)$ for the integer *c* such that all edges in $G[X\cup \{v\}]$ have weight *c*. If $n'$ is odd, |*X*| is even, and we can add exactly half its vertices to *C*. This way we have a cut $C''$ in $G[X \cup \{v\}]$ of weight at least

$$\begin{aligned} w(C'')&\ge c\Big (\frac{n'+1}{2}\Big )\Big (\frac{n'-1}{2}\Big )\\&= c\Big (\frac{n'(n'-1)}{4}\Big ) + c\Big (\frac{n'-1}{4}\Big )\\&= \frac{w(G[X \cup \{v\}])}{2} + \frac{w_{MSF}(G[X \cup \{v\}])}{4}. \end{aligned}$$

If $n'$ is even, we add $\frac{n'}{2}-1$ of the vertices of *X* to *C*, and leave $\frac{n'}{2}$ vertices out of *C*. In this case, we have a cut $C''$ in $G[X \cup \{v\}]$ of weight at least

$$\begin{aligned} w(C'')&\ge c\Big (\frac{n'}{2}\Big )\Big (\frac{n'}{2}\Big )\\&= c\Big (\frac{n'(n'-1)}{4}\Big ) + c\Big (\frac{n'}{4}\Big )\\&\ge \frac{w(G[X \cup \{v\}])}{2} + \frac{w_{MSF}(G[X \cup \{v\}])}{4}. \end{aligned}$$

In either case, we can see that we can combine $C'$ and $C''$ to a cut *C* in *G* of weight at least

$$\begin{aligned} w(C)&\ge \frac{w(G[X \cup \{v\}])}{2} + \frac{w_{MSF}(G[X \cup \{v\}])}{4} + \frac{w(G')}{2} + \frac{w_{MSF}(G')}{4} + \frac{k'}{4}\\&= \frac{w(G)}{2} + \frac{w_{MSF}(G)}{4} + \frac{k}{4}, \end{aligned}$$

where in the last equality we used that $w(G) = w(G[X \cup \{v\}]) + w(G')$, that $w_{MSF}(G) = w_{MSF}(G[X\cup \{v\}] + w_{MSF}(G')$, and that $k'=k$ for this rule.

***Rule*** 3: Since $G[X\cup \{v\}]$ is not uniform, we can apply Claim 15 to $G[X \cup \{v\}]$ to obtain a cut $C''$ in $G[X \cup \{v\}]$ of weight at least $\frac{w(G[X \cup \{v\}])}{2} + \frac{w_{MSF}(G[X \cup \{v\}])}{4} + \frac{1}{4}$. We now assume without loss of generality that $v\in C''\Leftrightarrow v\in C'$, i.e., both $C'$ and $C''$ put *v* on the same side of the cut. In this case we can combine $C'$ and $C''$ to a cut *C* of weight at least

$$\begin{aligned} w(C)&\ge \frac{w(G[X \cup \{v\}])}{2} + \frac{w_{MSF}(G[X \cup \{v\}])}{4} + \frac{1}{4} + \frac{w(G')}{2} + \frac{w_{MSF}(G')}{4} + \frac{k'}{4}\\&= \frac{w(G)}{2} + \frac{w_{MSF}(G)}{4} + \frac{k}{4}, \end{aligned}$$

where we used that $w_{MSF}(G) = w_{MSF}(G[X \cup \{v\}]) + w_{MSF}(G')$, that $k =k'+1$, and that $w(G) = w(G[X \cup \{v\}]) + w(G')$.

***Rule***4: We know that *v* must be adjacent to more than 1 and fewer than |*X*| vertices of *X*. We first do a case distinction on whether $G[X \cup \{v\}]$ is uniform or not.

If $G[X \cup \{v\}]$ is not uniform, we use the same argument as for the previous rule. Let $y \in X$ be a vertex not adjacent to *v*. Observe that for any $x\in X$ such that $\{v,x\}\in E$, $G[X \cup \{v\} - \{v,x\}]$ and $G[X \cup \{v\} - \{x,y\}]$ are both connected. Since $G[X\cup \{v\}]$ is not uniform but *G*[*X*] is, we can find such an *x* such that either $w(x,y) > w(x,v)$ or $w(x,y) < w(x,v)$. Therefore, we can use Claim 15 on $G[X \cup \{v\}]$. This gives us a cut $C''$ in $G[X\cup \{v\}]$ of weight at least $\frac{w(G[X \cup \{v\}])}{2} + \frac{w_{MSF}(G[X \cup \{v\}])}{4} + \frac{1}{4}$. Combining this cut with $C'$, we get a cut *C* in *G* of weight at least

$$\begin{aligned} w(C)&\ge \frac{w(G')}{2} + \frac{w_{MSF}(G')}{4} + \frac{k'}{4} + \frac{w(G[X \cup \{v\}])}{2} + \frac{w_{MSF}(G[X \cup \{v\}])}{4} + \frac{1}{4} \\&\ge \frac{w(G)}{2} + \frac{w_{MSF}(G)}{4} + \frac{k}{4}, \end{aligned}$$

where we used that $w(G) = w(G') + w(G[X \cup \{v\}])$, that $k = k' + 1$, and that $w_{MSF}(G) = w_{MSF}(G[X \cup \{v\}]) + w_{MSF}(G')$.

Otherwise $G[X \cup \{v\}]$ is *c*-uniform. Let $m'$ = $w(G[X\cup \{v\}])$ and $n'=|X|$. We can order the vertices in *X* as $x_1, x_2, \ldots , x_{n'}$ such that *v* is adjacent to exactly $x_1,\ldots ,x_r$, but not $x_{r+1},\ldots ,x_{n'}$. Assume without loss of generality that $v\in C'$. We add *v* and all $x_i$ for $i >\lceil \frac{n'}{2}\rceil$ to a cut $C''$ of $G[X\cup \{v\}]$. This cut has weight $s:=c(\lceil \frac{n'}{2} \rceil \cdot \lfloor \frac{n'}{2} \rfloor + \min \{r, \lceil \frac{n'}{2} \rceil \})$. Note that $m' = c(\frac{n'(n'-1)}{2} + r)$, thus we can rephrase *s*:

$$\begin{aligned} s&=\frac{m'}{2} - \frac{m'}{2} + c(\lceil \frac{n'}{2} \rceil \cdot \lfloor \frac{n'}{2} \rfloor + \min \{r, \lceil \frac{n'}{2} \rceil \}) \\&= \frac{m'}{2} + c(\frac{n'}{4}-\frac{n'^2}{4} -\frac{r}{2}) + c(\lceil \frac{n'}{2} \rceil \cdot \lfloor \frac{n'}{2} \rfloor + \min \{r, \lceil \frac{n'}{2} \rceil \})\\&= \frac{m'}{2} + c(\frac{n'}{4} - \frac{n'^2}{4} + \lceil \frac{n'}{2} \rceil \cdot \lfloor \frac{n'}{2} \rfloor + \min \{\frac{r}{2}, \lceil \frac{n'}{2} \rceil - \frac{r}{2}\}). \end{aligned}$$

If $n'$ is even, $\lceil \frac{n'}{2} \rceil \cdot \lfloor \frac{n'}{2} \rfloor = \frac{n'^2}{4}$, and then, similarly,

$$\begin{aligned} s\ge \frac{m'}{2} + c\frac{n'}{4} + \frac{c}{2} \ge \frac{m'}{2} + c\frac{n'}{4} + \frac{1}{4}. \end{aligned}$$

If $n'$ is odd, $\lceil \frac{n'}{2} \rceil \cdot \lfloor \frac{n'}{2} \rfloor = \frac{(n'+1)(n'-1)}{4} = \frac{n'^2}{4} - \frac{1}{4}$, and then

$$\begin{aligned} s\ge \frac{m'}{2} + c\frac{n'}{4} + \frac{c}{2} - \frac{c}{4} \ge \frac{m'}{2} + c\frac{n'}{4} + \frac{1}{4}. \end{aligned}$$

In either case we can combine $C''$ on $G[X\cup \{v\}]$ and $C'$ on $G'$ to get a cut *C* of *G* of weight at least

$$\begin{aligned} w(C)&\ge \frac{w(G')}{2} + \frac{w_{MSF}(G')}{4} + \frac{k'}{4} + \frac{m'}{2} + c\frac{n'}{4} + \frac{1}{4}\\&\ge \frac{w(G)}{2} + \frac{w_{MSF}(G)}{4} + \frac{k}{4}, \end{aligned}$$

where we used that an MSF of $G'$ can be turned into a spanning forest of *G* by adding $n'$ edges of weight *c*, so $w_{MSF}(G) \le w_{MSF}(G') + cn'$; that $k=k'+1$, and that $w(G) = w(G') + m'$.

***Rule*** 5: Let $X' = X - \{x,y\}$. If $w(x,v) > c$ or $w(y,v) > c$, since $G[X \cup \{v\} - \{v,x\}]$ and $G[X \cup \{v\} - \{v,y\}]$ are connected, we know by Claim 15 that $G[X \cup \{v\}]$ has a cut $C''$ of weight at least $\frac{w(G[X \cup \{v\}])}{2} + \frac{w_{MSF}(G[X \cup \{v\}])}{4} + \frac{1}{4}$. Since $G'$ and $G[X\cup \{v\}]$ overlap in only one vertex *v* we can w.l.o.g. assume that $v\in C''\Leftrightarrow v\in C'$, and we can combine $C''$ and $C'$ to a cut *C* of *G* of weight at least

$$\begin{aligned} w(C)&\ge \frac{w(G')}{2} + \frac{w_{MSF}(G')}{4} + \frac{k'}{4} +\frac{w(G[X \cup \{v\}])}{2} + \frac{w_{MSF}(G[X \cup \{v\}])}{4} + \frac{1}{4} \\&= \frac{w(G)}{2} + \frac{w_{MSF}(G)}{4} + \frac{k}{4}, \end{aligned}$$

where we used that $w(G) = w(G') + w(G[X \cup \{v\}])$, that $k = k' + 1$, and that $w_{MSF}(G) = w_{MSF}(G[X \cup \{v\}]) + w_{MSF}(G')$.

Thus, from now on we may assume $w(x,v) =w(y,v)= c$, i.e., the only edge in $G[X\cup \{v\}]$ that does not have weight *c* is the edge $\{x,y\}$ of weight $>c$. For the remaining cases, we perform a case distinction over the size of $X'$. Without loss of generality we assume that $v\not \in C'$.

- Case 1: $|X'|=1$. Let *u* be the only vertex in $X'$. Observe
  A1 $$\begin{aligned} w_{MSF}(G') + w(x,v) + w(y,v) + w(u,x) \ge w_{MSF}(G). \end{aligned}$$
  - Assume $w(x,y) > 2c$. We create *C* by only adding *x* to $C'$, i.e., $y,u\not \in C$. Then *C* has weight at least
    $$\begin{aligned} w(C)&\ge \frac{w(G')}{2} + \frac{w_{MSF}(G')}{4} + \frac{k'}{4} + w(x,y) + w(x,v) + w(x,u)\\&\ge \frac{w(G)}{2} + \frac{w_{MSF}(G')}{4} + \frac{k'}{4}\\  &\quad + \frac{w(x,y) + w(x,v)+w(x,u)-w(y,v)-w(y,u)}{2}\\&=\frac{w(G)}{2} + \frac{w_{MSF}(G')}{4} + \frac{k'}{4} + \frac{w(x,y)}{2}\\&>\frac{w(G)}{2} + \frac{w_{MSF}(G')}{4} + \frac{k'}{4} + \frac{2c}{2}\\&\ge \frac{w(G)}{2} + \frac{w_{MSF}(G')}{4} + \frac{k'}{4} + \frac{3c}{4} + \frac{1}{4}\\&\ge \frac{w(G)}{2} + \frac{w_{MSF}(G)}{4} + \frac{k}{4}, \end{aligned}$$
    where in the last inequality we used that $w_{MSF}(G') + 3c \ge w_{MSF}(G)$ by (A1) and $w(x,v) = w(y,v)=w(u,x)=c$, and that $k=k'+1$.
  - Assume $w(x,y) \le 2c$. We create *C* by adding only *x* and *y* to $C'$, i.e., $u\not \in C$. Then *C* has weight at least
    $$\begin{aligned} w(C)&\ge \frac{w(G')}{2} + \frac{w_{MSF}(G')}{4} + \frac{k'}{4} + w(y,v) + w(y,u) + w(x,v) + w(x,u) \\&\ge \frac{w(G')}{2} + \frac{w_{MSF}(G')}{4} + \frac{k'}{4} + 3c + \frac{w(x,y)}{2}\\&\ge \frac{w(G)}{2} + \frac{w_{MSF}(G')}{4} + \frac{k'}{4} + c\\&\ge \frac{w(G)}{2} + \frac{w_{MSF}(G)}{4} + \frac{k'}{4} + \frac{c}{4}\\&\ge \frac{w(G)}{2} + \frac{w_{MSF}(G)}{4} + \frac{k}{4}, \end{aligned}$$
    where we used that $w_{MSF}(G') + 3c \ge w_{MSF}(G)$ by (A1) and $w(x,v) = w(y,v)=w(u,x)=c$ in the penultimate step, and that $k' + c \ge k' + 1= k$ in the last step.
- Case 2: $$|X'|=:n'>1$$. Observe
  A2 $$\begin{aligned} w(G) = w(G') + c(\frac{n'(n'-1)}{2} + 2n'+2) + w(x,y) \end{aligned}$$
  and
  A3 $$\begin{aligned} w_{MSF}(G') + c(n'+2) \ge w_{MSF}(G). \end{aligned}$$
  - Assume $w(x,y) \ge 2c$. We start with a cut on $G[X']$ of weight at least $\frac{w(G[X'])}{2} + \frac{w_{MSF}(G[X'])}{4} = \frac{w(G[X'])}{2} + c(\frac{n'-1}{4})$ as guaranteed by the Poljak-Turzík bound. Then we extend this to a cut on $G[X'\cup \{x,y\}]$ by adding exactly one of *x* and *y*, choosing of the two possibilities the one that cuts at least half the weight in $E(X', \{x,y\})$. We combine this cut with the cut $C'$. The resulting cut *C* has weight at least
    $$\begin{aligned} w(C)&\ge \frac{w(G')}{2} + \frac{w_{MSF}(G')}{4} + \frac{k'}{4}  &   + \frac{w(G[X'])}{2} + c\Big (\frac{n'-1}{4}\Big )\\&\quad  &   + \frac{|E(X', \{x,y\})|}{2} + w(x,y) + c\\&\ge \frac{w(G)}{2} + \frac{w_{MSF}(G')}{4} + \frac{k'}{4}  &   + c\Big (\frac{n'-1}{4}\Big ) + \frac{w(x,y)}{2} \\&\ge \frac{w(G)}{2} + \frac{w_{MSF}(G')}{4} + \frac{k'}{4}  &   + c\Big (\frac{n'+3}{4}\Big )\\&\ge \frac{w(G)}{2} + \frac{w_{MSF}(G)}{4} + \frac{k'}{4}  &   + \frac{c}{4}\\&\ge \frac{w(G)}{2} + \frac{w_{MSF}(G)}{4} + \frac{k}{4}, \end{aligned}$$
    where in the second step we used that $w(G) = w(G') + w(G[X']) + E(X',\{x,y\}) + w(x,y) + 2c$, in the penultimate step we used (A3), and in the last step we used that $k' + c \ge k'+1 = k$.
  - Assume $w(x,y) < 2c$. We add both *x* and *y* to the cut $C'$. If $n'$ is odd we add $\frac{n'-1}{2}$ vertices of $X'$ to $C'$. The resulting cut *C* has weight at least
    $$\begin{aligned} w(C)&\ge \frac{w(G')}{2} + \frac{w_{MSF}(G')}{4} + \frac{k'}{4} + c\Bigg (\Big (\frac{n'+3}{2}\Big )\Big (\frac{n'+1}{2}\Big )+2\Bigg )\\&\ge \frac{w(G')}{2} + \frac{w_{MSF}(G')}{4} + \frac{k'}{4} + c\Big (\frac{n'^2+4n'+3}{4}+1\Big ) + \frac{w(x,y)+1}{2}\\&=\frac{w(G)}{2} + \frac{w_{MSF}(G')}{4} + \frac{k'}{4} + c\Big (\frac{n'-1}{4}+1\Big ) + \frac{1}{2}\\&= \frac{w(G)}{2} + \frac{w_{MSF}(G')}{4} + \frac{k'}{4} + c\Big (\frac{n'+3}{4}\Big ) + \frac{1}{2}\\&\ge \frac{w(G)}{2} + \frac{w_{MSF}(G)}{4} + \frac{k'}{4} + \frac{1}{2}\\&\ge \frac{w(G)}{2} + \frac{w_{MSF}(G)}{4} + \frac{k}{4}, \end{aligned}$$
    where in the third step we used (A2), in the penultimate step we used (A3), and in the last step we used $k' + 1 = k$. If $n'$ is even we add $\frac{n'}{2}-1$ vertices of $X'$ to $C'$. The resulting cut *C* has weight at least
    $$\begin{aligned} w(C)&\ge \frac{w(G')}{2} + \frac{w_{MSF}(G')}{4} + \frac{k'}{4} + c\Bigg (\Big (\frac{n'+2}{2}\Big )\Big (\frac{n'+2}{2}\Big )+2\Bigg ), \end{aligned}$$
    which is strictly larger than in the $n'$ odd case.

***Rule***6: Let $\min =\min (V {\setminus } \{a,b,c\},\{a,b,c\})$, and let $e_{\min }$ be an edge of weight $\min$ in $E(V{\setminus }\{a,b,c\},\{a,b,c\})$. Observe that $MSF(G')$ together with $e_{\min },\{a,b\}$, and $\{b,c\}$ forms a spanning forest of *G*. Therefore

A4 $$\begin{aligned} w_{MSF}(G') + \min + w(a,b) + w(b,c) = w_{MSF}(G') + \min + 2w(a,b) \ge w_{MSF}(G). \end{aligned}$$

We consider two subsets of $\{a,b,c\}$: $A_1=\{a,c\}$, and $A_2=\{b\}$. Considering these as cuts of *G*, both cuts cut the edges $\{a,b\}$ and $\{b,c\}$, and at least one of these cuts gets at least half of the total weight in $E(V(G'),\{a,b,c\})$. Enhancing $C'$ by that set, we therefore get a cut *C* of *G* of weight at least

$$\begin{aligned} w(C)&\ge \frac{w(G')}{2} + \frac{w_{MSF}(G')}{4} + \frac{k'}{4} + \frac{w(V(G'),\{a,b,c\})}{2} + w(a,b) + w(b,c) \\&\ge \frac{w(G)}{2} + \frac{w_{MSF}(G')}{4} + \frac{k'}{4} + \frac{w(a,b)}{2} + \frac{w(b,c)}{2}\\&\ge \frac{w(G)}{2} + \frac{w_{MSF}(G')}{4} + \frac{k'}{4} + \frac{w(a,b)}{2} + \frac{\min +1}{4}\\&\ge \frac{w(G)}{2} + \frac{w_{MSF}(G)}{4} + \frac{k'}{4} + \frac{1}{4}\\&\ge \frac{w(G)}{2} + \frac{w_{MSF}(G)}{4} + \frac{k}{4}, \end{aligned}$$

where in the second step we used that $w(G) = w(G') + w(V(G'),\{a,b,c\}) + w(a,b) + w(b,c)$, in the third step we used that $2w(b,c) > \min$, in the penultimate step we used (A4), and in the last step we used that $k'+1=k$.

***Rule*** 7: If *b* is adjacent to *v* we can augment $C'$ by adding *a*, *b*, *c* to *C* if and only if $v\not \in C'$. Observe $MSF(G') \cup \{a,v\} \cup \{b,v\} \cup \{c,v\}$ is a spanning forest of *G*, so

A5 $$\begin{aligned} w_{MSF}(G) \le w_{MSF}(G') + w(a,v) + w(b,v) + w(c,v). \end{aligned}$$

Also by the conditions of Rule 7 we have

A6 $$\begin{aligned} \frac{w(a,v)}{4} + \frac{w(b,v)}{4} \ge \frac{w(a,b)}{2} + \frac{w(a,b)}{2} = \frac{w(a,b)}{2} + \frac{w(b,c)}{2}. \end{aligned}$$

We can thus analyze the cut *C* to have weight at least

$$\begin{aligned} w(C)&\ge \frac{w(G')}{2} + \frac{w_{MSF}(G')}{4} + \frac{k'}{4} + w(a,v) + w(b,v) + w(c,v) \\&\ge \frac{w(G')}{2} + \frac{w_{MSF}(G')}{4} + \frac{k'}{4} + \frac{3w(a,v)}{4} + \frac{3w(b,v)}{4} + \frac{w(a,b)}{2} \\  &\quad + \frac{w(b,c)}{2} + w(c,v)\\&\ge \frac{w(G)}{2} + \frac{w_{MSF}(G')}{4} + \frac{k'}{4} + \frac{w(a,v)}{4} + \frac{w(b,v)}{4} + \frac{w(c,v)}{2}\\&\ge \frac{w(G)}{2} + \frac{w_{MSF}(G)}{4} + \frac{k'}{4} + \frac{w(c,v)}{4}\\&\ge \frac{w(G)}{2} + \frac{w_{MSF}(G)}{4} + \frac{k}{4}, \end{aligned}$$

where in the second step we used (A6), in the third step we used that $w(G) = w(G') + w(a,b) + w(b,c) + w(a,v) + w(b,v) + w(c,v)$, in the fourth step we used (A5), and in the last step we used that $k' + w(c,v) \ge k' + 1 = k$.

If *b* is not adjacent to *v*, add *a*, *c* to *C* if and only if $v\not \in C'$, and we add *b* to *C* if and only if $v\in C'$. Thus the edges $\{a,b\},\{b,c\},\{a,v\},\{c,v\}$ are all cut. Note that $MSF(G')\cup \{c,v\}\cup \{a,b\}\cup \{b,c\}$ is a spanning forest of *G*, so

A7 $$\begin{aligned} w_{MSF}(G) \le w_{MSF}(G') + w(c,v) + w(a,b) + w(b,c). \end{aligned}$$

The cut *C* has weight at least

$$\begin{aligned} w(C)&\ge \frac{w(G')}{2} + \frac{w_{MSF}(G')}{4} + \frac{k'}{4} + w(a,v) + w(a,b) + w(b,c) + w(c,v)\\&\ge \frac{w(G)}{2} + \frac{w_{MSF}(G')}{4} + \frac{k'}{4} + \frac{w(a,v)}{2} + \frac{w(c,v)}{2} + \frac{w(a,b)}{2} + \frac{w(b,c)}{2}\\&\ge \frac{w(G)}{2} + \frac{w_{MSF}(G)}{4} + \frac{k'}{4} + \frac{w(a,v)}{2} + \frac{w(c,v)}{4} + \frac{w(a,b)}{4} + \frac{w(b,c)}{4}\\&\ge \frac{w(G)}{2} + \frac{w_{MSF}(G)}{4} + \frac{k}{4}, \end{aligned}$$

where in the second step we used that $w(G) = w(G') + w(a,v) + w(a,b) + w(b,c) + w(c,v)$, in the third step we used (A7), and in the last step we used that $k' + w(a,v) \ge k' + 1 =k$.

***Rule*** 8: Let *v* be the only neighbor of $\{x,y\}$ in *Y* and let $\overline{n} = |X|$. We first extend $C'$ to $C''$ by adding *x*, *y* to $C''$ if and only if $v\not \in C'$. We then extend $C''$ to *C* as follows.

Without loss of generality, assume $x,y\not \in C''$. We perform a case distinction on the parity of $\overline{n}$. Note that $w(G[X \cup \{x,y\}]) = c(\frac{\overline{n}(\overline{n}-1)}{2} + 2\overline{n})$.

- If $\overline{n}$ is odd, we add $\frac{\overline{n}+1}{2}$ of the vertices in *X* to *C*. In $G[X\cup \{x,y\}]$ this cuts in total a weight of
  $$\begin{aligned} c\Bigg (\Big (\frac{\overline{n}+1}{2}\Big )\Big (\frac{\overline{n}-1}{2}\Big ) + 2\frac{\overline{n}+1}{2}\Bigg )&= c\Big (\frac{\overline{n}(\overline{n}-1)}{4} + \frac{\overline{n}-1}{4} + \overline{n} + 1\Big )\\&= \frac{w(G[X \cup \{x,y\}])}{2} + c\Big (\frac{\overline{n}}{4} + \frac{3}{4}\Big ). \end{aligned}$$
- If $\overline{n}$ is even, we add $\frac{\overline{n}}{2} + 1$ vertices in *X* to *C*. In $G[X\cup \{x,y\}]$ this cuts in total a weight of
  $$\begin{aligned} c\Bigg (\Big (\frac{\overline{n}}{2} + 1\Big )\Big (\frac{\overline{n}}{2}-1\Big ) + 2\Big (\frac{\overline{n}}{2}+1\Big )\Bigg )&= c\Big (\frac{\overline{n}^2}{4} + \overline{n} + 1\Big ) \\ = c\Big (\frac{\overline{n}^2}{4} + \frac{3}{4}\overline{n} + \frac{\overline{n}}{4} + 1\Big )&= \frac{w(G[X \cup \{x,y\}])}{2} + c\Big (\frac{\overline{n}}{4} + 1\Big ). \end{aligned}$$

In either case we thus have that *C* cuts at least half of the weight in $G[X\cup \{x,y\}]$ plus $c(\frac{\overline{n}}{4}+\frac{3}{4})$.

Observe that

A8 $$\begin{aligned} w_{MSF}(G) \le w_{MSF}(G') + c\overline{n} + w(x,v) + w(y,v). \end{aligned}$$

In total we can thus bound the weight of the cut *C* as

$$\begin{aligned} w(C)&\ge \frac{w(G')}{2} + \frac{w_{MSF}(G')}{4} + \frac{k'}{4} + \frac{w(G[X \cup \{x,y\}])}{2} + c(\frac{\overline{n}}{4}\\  &\quad + \frac{3}{4}) + w(x,v) + w(y,v)\\&= \frac{w(G)}{2} + \frac{w_{MSF}(G')}{4} + \frac{k'}{4} + c(\frac{\overline{n}}{4} + \frac{3}{4}) + \frac{w(x,v)}{2} + \frac{w(y,v)}{2}\\&\ge \frac{w(G)}{2} + \frac{w_{MSF}(G)}{4} + \frac{k'}{4} + \frac{3c}{4} + \frac{w(x,v)}{4} + \frac{w(y,v)}{4}\\&\ge \frac{w(G)}{2} + \frac{w_{MSF}(G)}{4} + \frac{k}{4}, \end{aligned}$$

where in the second step we used $w(G) = w(G') + w(G[X \cup \{x,y\}) + w(x,v) + w(y,v)$, in the third step we used (A8), and in the last step we used that $k' + w(x,v) \ge k' + 1 = k$.

We conclude that for every rule, from a cut $C'$ of $G'$ of the guaranteed weight we can build a cut *C* of *G* of the required weight, and thus the lemma follows. $\square$

## Footnotes

1 For MaxCut this property would be bipartiteness.
